# O078: 6 years of national German hand hygiene campaign-where do we come from and where are we heading to?

**DOI:** 10.1186/2047-2994-2-S1-O78

**Published:** 2013-06-20

**Authors:** C Reichardt, M Behnke, K Bunte-Schönberger, P Gastmeier

**Affiliations:** 1 Institute of Hygiene, Universitiy Medicine Berlin, Charite, Berlin, Germany

## Introduction

The national German hand hygiene (HH) campaign “AKTION Saubere Hände” started at January 1^st^ 2008. The campaign is based on the WHO “Clean Care is Safer Care” campaign and is funded for six years be the German ministry of health. By March 2013, over 1300 health care institutions are actively participating.

## Objectives

We present data on the development of observed compliance rates, alcohol based hand rub consumption (AHC) and hand rub dispenser availability over the campaign period.

## Methods

All participants have to implement a multimodal intervention program. Among other parameters, they have to collect the following data: AHC, hand rub dispenser availability and on a voluntary basis observed compliance data. All three parameters are collected using a defined data collection tool. Observers are trained by the campaign team members. AHC is given unit based yearly in ml per patient day. Hand rub availability is defined as one dispenser per ICU bed and per two non-ICU beds. The definition of HH opportunities (HHO) is based on the WHO Model “My 5 moments of hand hygiene”. Observations were done before intervention and after interventions. A minimum of 200 observations per unit was defined.

## Results

There are 1300 health care institutions participating in the campaign by March 2013. Among those are 815 hospitals (45% of all hospitals in Germany), 66 rehabilitation clinics, 242 long term care facilities and 183 outpatient care facilities. There was an increase of AHC of 47,5% (p<0,001) in 166 hospitals that provided AHC data consecutively from 2007 to 2011. Hand rub dispenser availability increased to over 90% in non-ICU’s and to over 100% in ICU’s (p<0,001). Compliance increased from 64% before to 74% after one intervention in 82 hospitals with 249 units (136205 HHO’s) (P<0,001). 6 hospitals with 25 units have observed over four periods. Observed HH compliance increased from 60% at baseline to 71,2% after four observation periods (p<0,001).

## Conclusion

Hospitals participating in the campaign have to implement a multimodal intervention program. The increase of AHC and observed compliance show, that the multimodal intervention program based on the WHO strategy led to improved HH compliance.

## Disclosure of interest

None declared.

